# Screening for obstructive sleep apnea among hospital outpatients

**DOI:** 10.1371/journal.pone.0198315

**Published:** 2018-05-30

**Authors:** Michel Hug, Katrin Uehli, Stig Solbach, Stefanie Brighenti-Zogg, Selina Dürr, Sabrina Maier, Jörg Daniel Leuppi, David Miedinger

**Affiliations:** 1 Faculty of Medicine, University of Basel, Basel, Switzerland; 2 Swiss National Accident Insurance Fund (SUVA), Lucerne, Switzerland; 3 University Clinic of Medicine, Cantonal Hospital Baselland, Liestal, Switzerland; Charité - Universitätsmedizin Berlin, GERMANY

## Abstract

**Background:**

Obstructive sleep apnea syndrome (OSAS) is common in adults. People with OSAS have a higher risk of experiencing traffic accidents and occupational injuries (OIs). We aimed to clarify the diagnostic performance of a three-channel screening device (ApneaLink^TM^) compared with the gold standard of full-night attended polysomnography (PSG) among hospital outpatients not referred for sleep-related symptoms. Furthermore, we aimed to determine whether manual revision of the ApneaLink^TM^ autoscore enhanced diagnostic performance.

**Methods:**

We investigated 68 patients with OI and 44 without OI recruited from the University Hospital Basel emergency room, using a cross-sectional study design. Participating patients spent one night at home with ApneaLink^TM^ and within 2 weeks slept for one night at the sleep laboratory. We reanalyzed all ApneaLink^TM^ data after manual revision.

**Results:**

We identified significant correlations between the ApneaLink^TM^ apnea-hypopnea index (AHI) autoscore and the AHI derived by PSG (*r* = 0.525; *p* <0.001) and between the ApneaLink^TM^ oxygen desaturation index (ODI) autoscore and that derived by PSG (*r* = 0.722; *p* <0.001). The ApneaLink^TM^ autoscore showed a sensitivity and specificity of 82% when comparing AHI ≥5 with the cutoff for AHI and/or ODI ≥15 from PSG. In Bland Altman plots the mean difference between ApneaLink^TM^ AHI autoscore and PSG was 2.75 with SD ± 8.80 (β = 0.034), and between ApneaLink^TM^ AHI revised score and PSG -1.50 with SD ± 9.28 (β = 0.060).

**Conclusions:**

The ApneaLink^TM^ autoscore demonstrated good sensitivity and specificity compared with the gold standard (full-night attended PSG). However, Bland Altman plots revealed substantial fluctuations between PSG and ApneaLink^TM^ AHI autoscore respectively manually revised score. This spread for the AHI from a clinical perspective is large, and therefore the results have to be interpreted with caution. Furthermore, our findings suggest that there is no clinical benefit in manually revising the ApneaLinkTM autoscore.

## Introduction

Obstructive sleep apnea syndrome (OSAS) is characterized by repetitive episodes of upper airway obstruction during sleep, which is associated with intermittent hypoxemia, increased work in breathing, and awakening [1]. In general, OSAS is caused by anatomical and/or functional instability of the upper airway, potentially leading to upper airway collapse. Risk factors contributing to OSAS are pre-existing snoring (years of snoring can lead to neurodegenerative damage from vibration trauma and foster an obstruction of the muscular tube) [2] or increasing tissue pressure (e.g. due to adiposity). Studies have demonstrated that elevated body mass index (BMI) can accelerate the development of OSAS or the severity of apnea over time [3]. Snoring may be the earliest nocturnal symptom of OSAS [4]. OSAS is common with a prevalence of at least 4% among the middle-aged male Caucasian population [5,6].

The most common clinical symptom of OSAS is excessive daytime sleepiness [5,7], because of the significant reduction of continuous sleep duration and efficiency. A previous study showed that excessive daytime sleepiness affects 7% of the Western population [8]. This daytime sleepiness may be a consequence of sleep-associated breathing disorders [9–11]. Furthermore, multiple studies reported an increased prevalence of OSAS in patients with heart failure [12,13], hypertension [14,15], cardiovascular diseases [16,17], cerebrovascular diseases [18], and insulin resistance [19,20].

The American Academy of Sleep Medicine (AASM) recommends PSG [21] to diagnose suspected OSAS. However, the high prevalence of individuals with OSAS risk factors and the limited capacity/availability [22] of sleep laboratories present problems. It is estimated that 82% of men and 93% of women with OSAS remain undiagnosed [23,4].

Reaction time, skills, and a good psycho-physical capacity diminish during the normal working day. Breaks and sleep are fundamental for relaxation and to regain capacity [24]. The odds ratio for fatigue at work was 30 times higher in heavy snorers and 10 times higher in patients with OSAS compared with reference individuals [11].

Official reports indicate excessive daytime sleepiness is a primary reason for road traffic accidents, which are often associated with significant morbidity and mortality [25]. Moreover, increasing age, fatigue, smoking, sleep disorders, and conditions affecting balance increase the risk of injury or repeated injury [26–34].

Screening for OSAS and sleep disturbances in the general workforce is rarely performed. In the industrial sector, key reasons for the lack of OSAS screening among employees are the large financial outlay required, few hospitals with OSAS specialists, few laboratories for evaluation, and little interest from management [35]. However, screening for sleep-related breathing disorders could be performed when workers with occupational injury (OI) present to emergency care. In this context, a readily available screening device could be used in the days following injury.

Unlike other studies, this study aimed to investigate the diagnostic performance of ApneaLink^TM^, a three-channel apnea screening device, in two groups of individuals with a relatively low risk of OSAS in an ambulatory setting, although AASM recommends screening only for people with a high pretest probability. We compared ApneaLink^TM^ diagnoses of nocturnal apneas, hypopneas, and desaturations with those measured with full-night attended PSG, currently the gold standard. Furthermore, we investigated whether a manual review and scoring of ApneaLink^TM^ recordings would increase diagnostic validity compared with the PSG.

## Materials and methods

Written informed consent regarding the procedures and for the medical data to be used was obtained from all patients according to the guidelines of the local Ethics Regulation Committee of Basel named Ethikkommission beider Basel (EKBB). Due to a regional expansion in 2015 they changed the name from EKBB to Ethikkommission Nordwest- und Zentralschweiz (EKNZ). The review board of the commission approved this protocol in accordance with the amended Declaration of Helsinki (reference number: 37/09).

We investigated 68 patients with an OI who presented to the University Hospital Basel emergency room and 44 patients without OI who visited the hospital's outpatient department for other reasons. Patients were recruited between December, 2009 and March, 2012. Recruitment was done on at least one to two working days (Monday-Friday) during a week. On these days, recruitment was consecutive. Height and weight were measured, and BMI was calculated for all participating patients. We included patients aged between 18–65 years, with a theoretical work capacity of 100% (= the person could work at least 42 hours per week) and an actual capacity of at least 50% of full-time employment, as a lower employment does not seem to be sufficient for representing a meaningful time period. We excluded patients with injuries to the brain, thorax, or spinal cord.

After receiving required medical care, patients who had provided written informed consent spent the following night at home with the ApneaLink^TM^ device. A study nurse instructed patients regarding use of ApneaLink^TM^.

### ApneaLink^TM^ device [36]

ApneaLink^TM^ is a multichannel screening tool developed by ResMed that measures nasal respiratory flow, blood oxygen saturation, and pulse rate. The device is strapped to the chest with a belt to ensure the device is aligned with the nasal tube and the pulse oximetry channel. Using a nasal pressure transducer, breath sounds and nasal respiratory flow are registered. The connection between the nose and the device ensures a breath flexible hise. A luer connector—a standardized connection system for hose system in the medical field—assures the connection between the device and the hose. The blood oxygen saturation and pulse rate are detected by a ApneaLink^TM^ oximeter and a finger pulse detector. The finger pulse detector measures oxygen saturation in peripheral blood by using red light rays, that it emits through the skin of the fingertip. ApneaLink^TM^ weighs only 50g and is 12.5cm x 6cm x 3cm (length x width x height) in size.The device is battery powered and has a storage capacity of 15 megabytes, corresponding to a minimum of 10-h recording time. The included software analyzes signal-channel created data and produces a report in PDF format. The one-sided standard report shows all the details of the nocturnal recording and analysis. We used ApneaLink^TM^ Software version 8.00 and Firmware version 4.08. Patients returned the ApneaLink^TM^ device after one night, and we imported the resulting data into our computer.

To control ApneaLink^TM^ performance, all data were evaluated manually by a medical assistant after receiving detailed instructions from the head of the University Hospital Basel sleep laboratory. The preinstalled ApneaLink^TM^ definitions for all parameters were adopted. During manual analysis, the medical assistant was blinded to PSG outcomes and to group assignment of patients. All reviews were performed by the same person.

We focused on the apnea-hypopnea index (AHI) and the oxygen desaturation index (ODI). The AHI is the number of apneas (obstructive, central, and mixed type) and hypopneas per hour of valid recorded sleep time. The ODI indicates the number of blood oxygen desaturations per hour of evaluable sleep time. The first 10 min are automatically excluded from evaluation by the ApneaLink^TM^ software (to consider a delay of sleep). The end of the evaluation and time slots for invalid or missing signal data are also excluded. A minimum of 180 valid minutes of recorded nasal flow signal and/or pulse oximetry signal was required to be included in our analysis.

### Full-night attended polysomnography (PSG)

Within 2 weeks after using ApneaLink^TM^, the OI patient group slept for one night in the sleep laboratory at the University Hospital Basel. All participants underwent full-night attended PSG. A trained medical assistant monitored participants and recordings during the night to ensure continuous recording of data. We recorded chest and abdomen movements, nasal flow, pulse oximetry, electrocardiogram, submental and tibialis electromyogram, electroencephalogram, and electrooculogram, accompanied by a microphone and infrared camera for monitoring. Sleep data were analyzed by a trained physician qualified and experienced in the analysis of sleep studies. PSG recordings were performed according to the AASM Manual for Scoring Sleep and Associated Events criteria. A hypopnea was defined according to AASM as respiratory flow reduced by at least 30% for ≥ 10 seconds, or reduction in respiratory flow with desaturation accompanied by at least 4%. The maximum duration of a hypopnea was set to 100 seconds. PSG data were collected using RemLogic PSG software (Embla, Broomfield CO, USA).

### Statistical analysis

We used IBM SPSS Statistics version 22 for Apple Macintosh for the statistical analyses. Descriptive statistics were computed (mean and standard deviation) for continuous variables. The significance level was set at *p* < 0.05. The Shapiro-Wilk test was used to examine whether data were normally distributed. Moreover, we visually assessed histograms.

Performance of the ApneaLink^TM^ device compared to PSG was evaluated using sensitivity and specificity analysis. Furthermore, we used Spearman's rho coefficient, to evaluate correlations and chi-square tests to test significant differences between patients with and without OI.

## Results

In total 160 patients agreed to participate in this study; 112 patients (70%) underwent clinical examination and completed ApneaLink^TM^ and PSG investigations.

**Fig 1:**
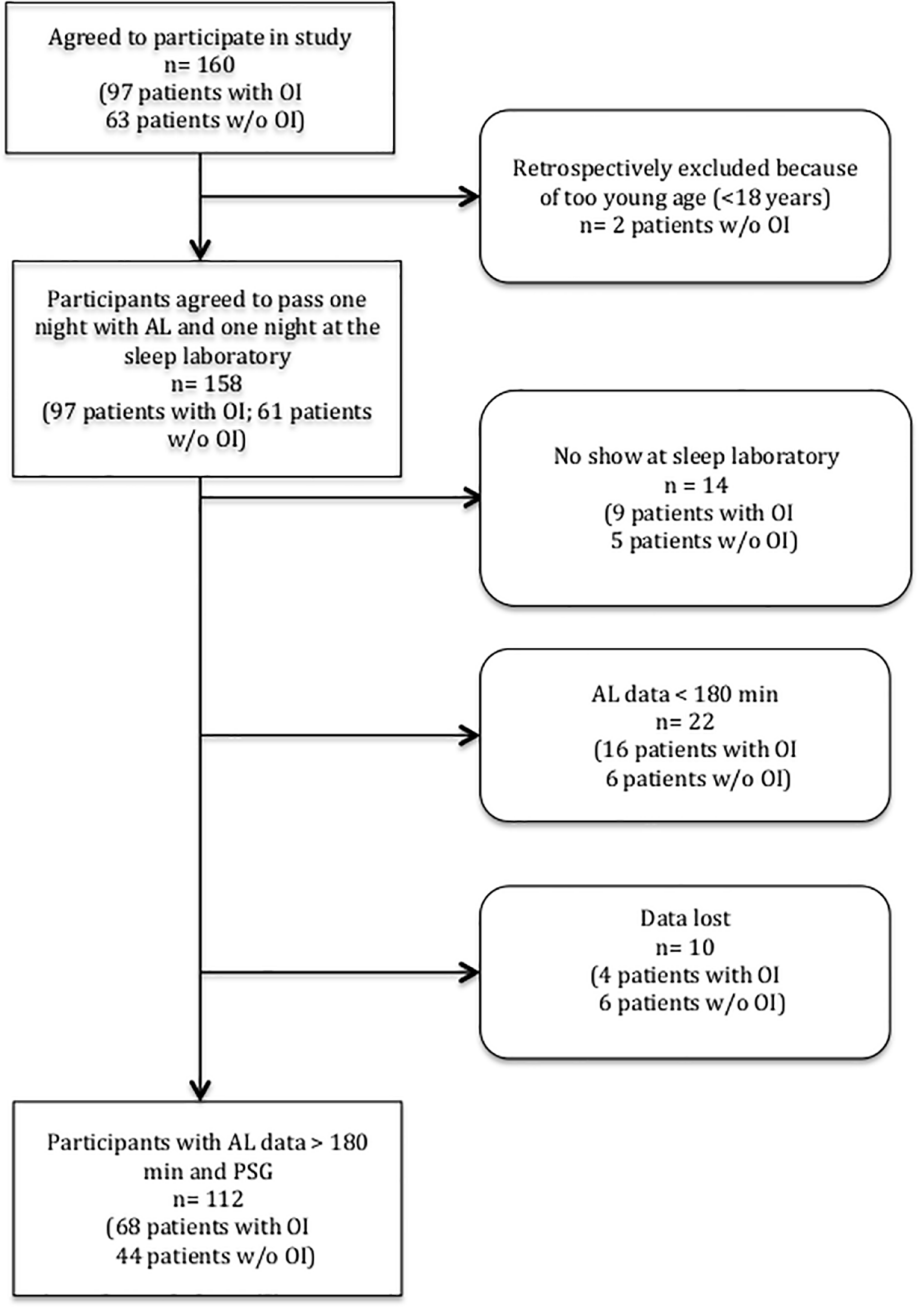
Flow of study participants. OI, occupational injury; w/o, without; PSG, polysomnography.

Forty-six patients (29%) did not attend the sleep laboratory nor did not have the minimum ApneaLink^TM^ recording time of 180 min. Fig 1 illustrates the flow of study participants. Descriptive statistics are presented in Table 1.

**Table 1:** Demographic and clinical characteristics of study participants.

Variables	Total (*n* = 112)	Patients with OI (*n* = 68)	Patients w/o OI (*n* = 44)
	M (SD)	M (SD)	M (SD)
**Age (years)**	40 (13)	39 (14)	41 (12)
**BMI (kg/m**^**2**^**)**	26 (5)	26 (5)*	24 (4)*
**Gender (f/m)**	52 (46%)/61 (54%)	29 (43%)/39 (57%)	22 (50%)/22 (50%)

BMI, body mass index; OI, occupational injury; w/o, without; M, mean; SD, standard deviation; f, female; m, male; *Significant difference between patients with and w/o OI using Mann Whitney test (p = 0.045, 2-sided).

### Number of evaluable measurements

A minimum recording time of 180 min was required from either the pulse oximetry channel or the nasal flow channel of the ApneaLink^TM^ to fulfill eligibility criteria. A minimum of 180 min of pulse oximetry measurement was available in 88% of patients (mean recording time 410 ± 98 min); and after manual review of ApneaLink^TM^ data this information was available for 87% (mean recording time 409 ± 96 min). In nine (8%) patients, the manual review changed the total number of minutes of pulse oximetry data. In two (2%) of these patients, the extra time gained was ≥60 min. The time slot for invalid or missing pulse oximetry signal data for participants included into the study was 0.9% (376 min of total 40131 min).

A minimum of 180 min of nasal flow measurement was available in 88% of patients (mean recording time 373 ± 115 min). Manual review of the ApneaLink^TM^ data yielded nasal flow data for 92% of patients (mean recording time 381 ± 113 min). In 41 patients (37%), the manual review changed the total number of minutes of nasal flow data, and in nine (8%) patients, the extra time gained was ≥60 min. The time slot for invalid or missing nasal flow signal data for participants included into the study was 1.3% (493 min of total 37504 min).

### Correlations between ApneaLink^TM^ and PSG

Table 2 shows the AHI and ODI determined by ApneaLink^TM^ (autoscore and after manual revision) compared with AHI and ODI derived from PSG (Spearman's rho correlation coefficients with *p*-values).

**Table 2:** Correlations between ApneaLink^TM^ and polysomnography.

Spearman's rho, r	AHI AL as	AHI AL re	AHI PSG	ODI AL as	ODI AL re	ODI PSG
**AHI AL as**	-					
**AHI AL re**	*r* = 0.926, *p* <0.001	-				
**AHI PSG**	*r* = 0.525, *p* <0.001	*r* = 0.548, *p* <0.001	-			
**ODI AL as**	*r* = 0.831, *p* <0.001	*r* = 0.825, *p* <0.001	*r* = 0.569, *p* <0.001	-		
**ODI AL re**	*r* = 0.821, *p* <0.001	*r* = 0.801, *p* <0.001	*r* = 0.549, *p* <0.001	*r* = 0.951, *p* <0.001	-	
**ODI PSG**	*r =* 0.679, *p* <0.001	*r* = 0.686, *p* <0.001	*r* = 0.738, *p* <0.738	*r* = 0.722, *p* <0.001	*r* = 0.712, *p* <0.001	-

r, Spearman-Rho correlation coefficient; as, autoscore; re, revised score; *p*, p-value; AHI, apnea-hypopnea index; AL, ApneaLink^TM^; PSG, polysomnography; ODI, oxygen desaturation index.

High correlations were found between the ApneaLink^TM^ autoscore and revised data for both AHI (*r* = 0.926, *p* <0.001) and ODI (*r* = 0.951, *p* <0.001). Moreover, high correlations were found between the ODI and AHI ApneaLink^TM^ autoscores (*r* = 0.831, *p* <0.001). Furthermore, significant correlations were detected in ODI between the ApneaLink^TM^ autoscore and PSG (*r* = 0.722, *p* <0.001) and between the AHI ApneaLink^TM^ autoscore and ODI derived by PSG (*r* = 0.679, *p* <0.001).

**Fig 2:**
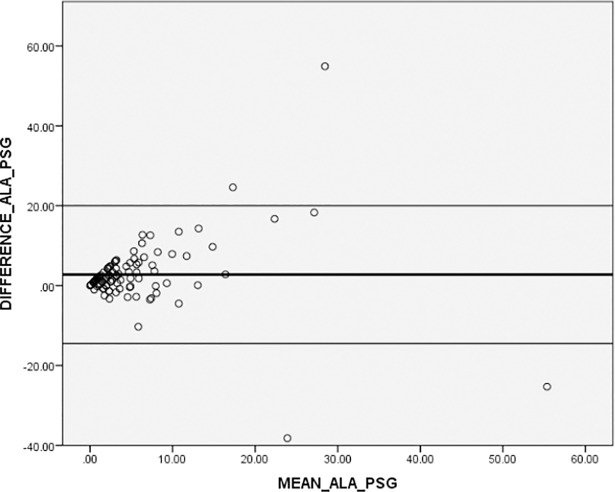
Bland-Altman plot illustrating the difference in AHI as measured from ApneaLink^TM^ autoscore versus PSG. **Plots present differences between the two methods compared to mean AHI of the two methods. The black solid line represents the mean difference, the black thin lines the 95% confidence intervals on the limits of agreement.** ALA, ApneaLink^TM^ autoscore; PSG, polysomnography.

**Fig 3:**
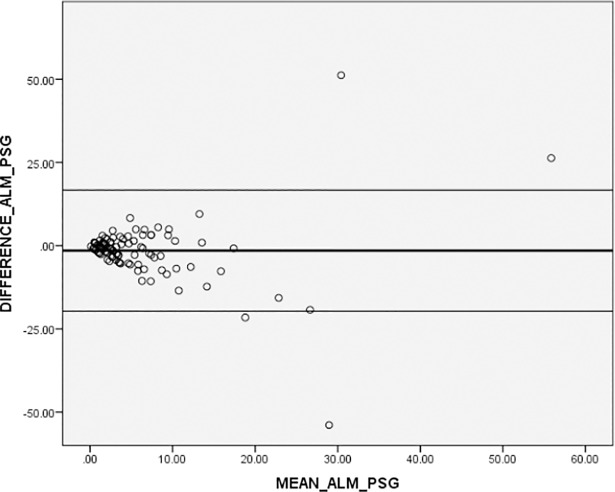
Bland-Altman plot illustrating the difference in AHI as measured from ApneaLink^TM^ manually revised versus PSG. **Plots present differences between the two methods compared to mean AHI of the two methods. The black solid line represents the mean difference, the black thin lines the 95% confidence intervals on the limits of agreement.** ALM, ApneaLink^TM^ manually revised; PSG, polysomnography.

Figs 2 and 3 present Bland-Altman plots in addition to the correlations for the AHI. When comparing the ApneaLink^TM^ autoscore to PSG, the mean difference was 2.75 with SD ± 8.80 (β = standardized coefficient = 0.034), and for ApneaLink^TM^ revised score vs PSG, a mean difference of -1.50 with SD ± 9.28 (β = 0.060) was detected.

The AHI and ODI from patients with recorded ApneaLink^TM^ data ≤180 minutes did not correlate significantly with PSG results (data not shown).

In Table 3, the rate of misclassifications of OSAS diagnosis by using variable AHI thresholds (<5, 5–15, >15) are provided.

**Table 3:** Rate of misclassifications of OSAS diagnosis between ApneaLink^TM^ autoscore, ApneaLink^TM^ manually revised score and polysomnography.

	AHI < 5 PSG	AHI 5–15 PSG	AHI >15 PSG
**AHI AL autoscore**	10%	62.1%	80%
**AHI AL revised**	17.2%	44.8%	70%

AHI, apnea-hypopnea index; AL, ApneaLink^TM^; PSG, polysomnography.

### Sensitivity, specificity, positive predictive value, and negative predictive value of ApneaLink^TM^

The diagnostic performance of ApneaLink^TM^ autoscores and ApneaLink^TM^ after manual revision is shown in Tables 4 and 5.

**Table 4:** Sensitivity, specificity, positive predictive value and negative predictive value of ApneaLink^TM^ autoscore compared with polysomnography.

ApneaLink^TM^ Autoscore	PSG AHI ≥5				PSG AHI and/or ODI ≥5				PSG AHI and/or ODI ≥15				Missing (%)
	S	Sp	PPV	NPV	S	Sp	PPV	NPV	S	Sp	PPV	NPV	
**AHI ≥5**	49%	90%	76%	73%	50%	92%	80%	73%	82%	82%	36%	97%	13 (12%)
**AHI and/or ODI ≥5**	48%	89%	73%	74%	50%	91%	77%	74%	78%	81%	32%	97%	24 (21%)
**AHI and/or ODI ≥15**	12%	98%	80%	65%	12%	98%	80%	64%	44%	99%	80%	94%	24 (21%)

AHI, apnea-hypopnea index; ODI, oxygen desaturation index; S, sensitivity; Sp, specificity; PPV, positive predictive value; NPV, negative predictive value; PSG, polysomnography.

**Table 5:** Sensitivity, specificity, positive predictive value, and negative predictive value of ApneaLink^TM^ after manual revision compared with polysomnography.

ApneaLink^TM^ revised	PSG AHI ≥5				PSG AHI and/or ODI ≥5				PSG AHI and/or ODI ≥15				Missing (%)
	S	Sp	PPV	NPV	S	Sp	PPV	NPV	S	Sp	PPV	NPV	
**AHI ≥5**	64%	83%	69%	79%	65%	84%	72%	79%	82%	71%	25%	97%	9 (8%)
**AHI and/or ODI ≥5**	61%	82%	67%	78%	71%	84%	70%	78%	78%	71%	23%	97%	23 (21%)
**AHI and/or ODI ≥15**	12%	98%	80%	65%	12%	98%	80%	64%	33%	98%	60%	93%	23 (21%)

AHI, apnea-hypopnea index; ODI, oxygen desaturation index; S, sensitivity; Sp, specificity; PPV, positive predictive value; NPV, negative predictive value; PSG, polysomnography.

The highest sensitivity (82%) for both the ApneaLink^TM^ autoscore and after manual revision was found when comparing AHI ≥5 with PSG AHI and/or ODI cutoff ≥15. The highest specificity (99% vs. 98% after manual revision) was found for AHI and/or ODI ≥15 compared with PSG AHI and/or ODI ≥15. The ApneaLink^TM^ autoscore showed the highest positive predictive value (PPV; 80%) for AHI and/or ODI ≥15 compared with the PSG AHI and/or ODI cutoff ≥15. After ApneaLink^TM^ manual revision, the highest PPV (80%) was found when comparing AHI and/or ODI ≥15 with the PSG AHI and/or ODI cutoff ≥5. The highest negative predictive value (NPV) of the ApneaLink^TM^ autoscore (also after manual revision) was 97% when comparing AHI ≥5 with PSG AHI and/or ODI ≥15.

The Likelihood ratio of a positive test (LR+) shows for ApneaLink^TM^ autoscore AHI ≥5 a LR+ of 3.76 (pretest probability 39.4%, posttest probability 70.9%) and for ApneaLink^TM^ manually revised AHI ≥5 a LR+ of 3.73 (pretest probability 38.0%, posttest probability 69.6%) compared to PSG. For ApneaLink^TM^ autoscore AHI ≥15, a LR+ of 18.18 (pretest probability 10.0%, posttest probability 66.9%) was detected, and for ApneaLink^TM^ manually revised AHI ≥15 a LR+ of 13.64 (pretest probability 9.7%, posttest probability 60.0%).

### Comparison of AHI between ApneaLink^TM^ (autoscore and manually revised) and PSG

**Fig 4:**
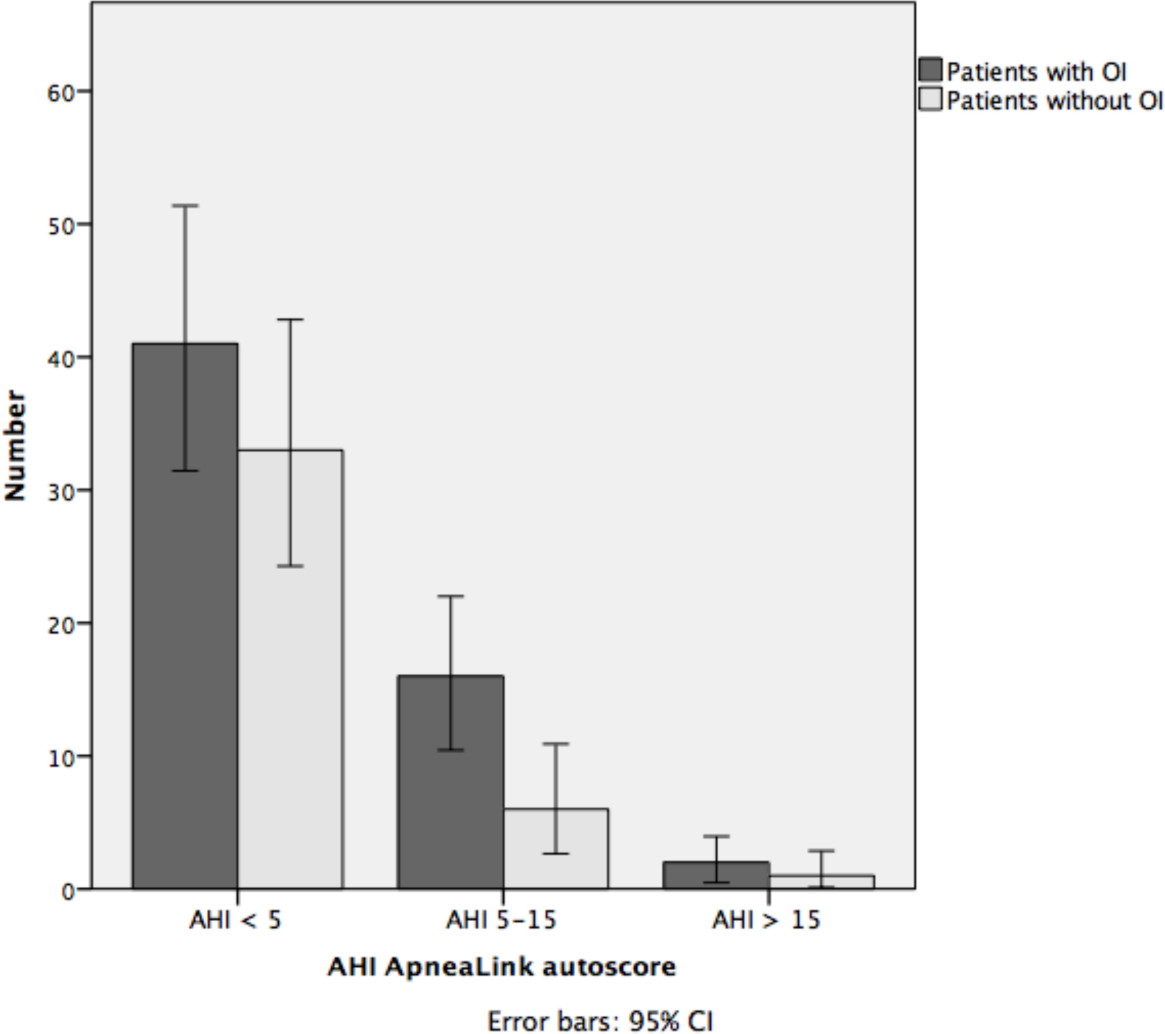
Distribution of patients with and without an OI across AHI categories based on ApneaLink^TM^ autoscore. OI, occupational injury; AHI, apnea-hypopnea index; CI, confidence interval.

**Fig 5:**
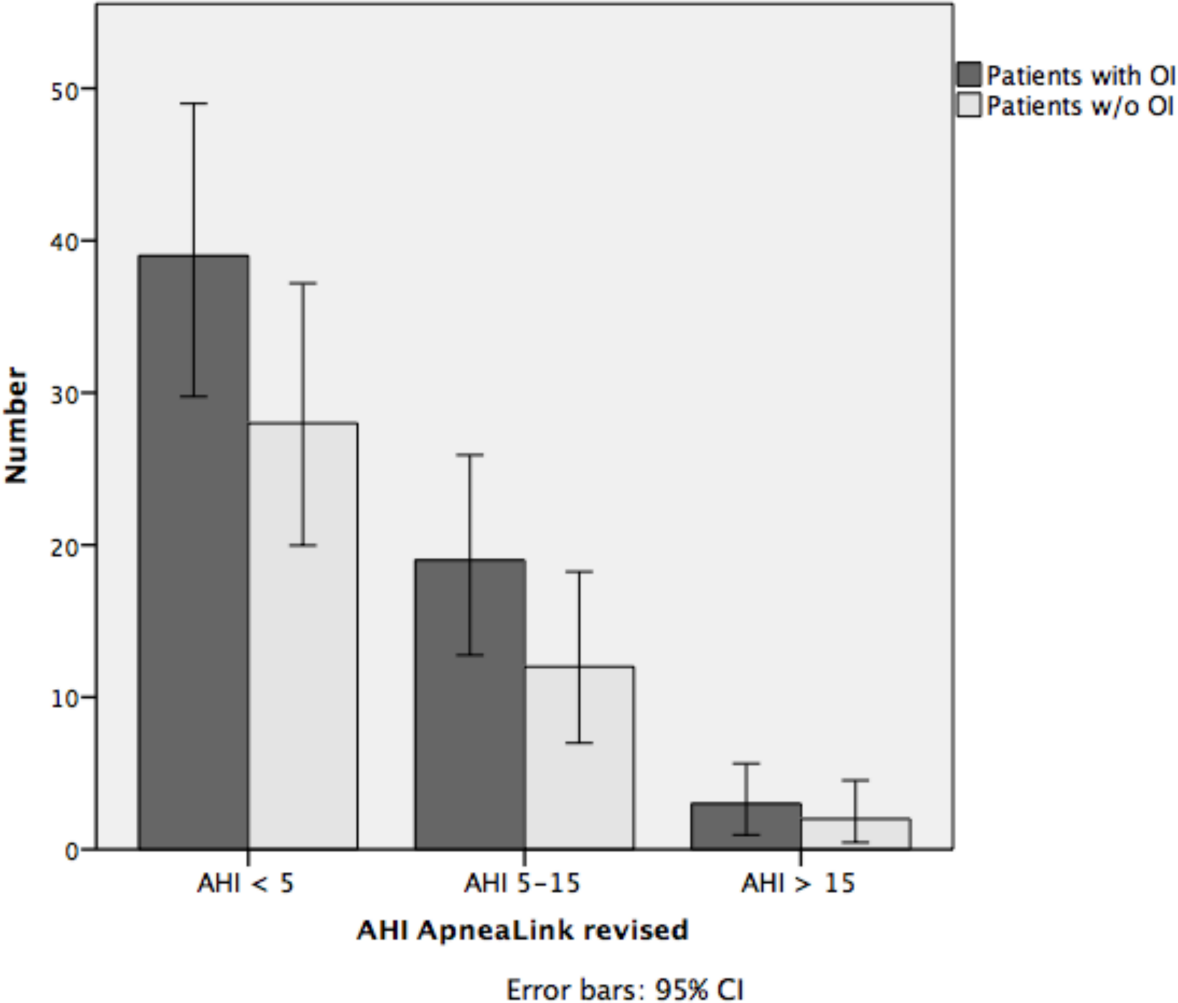
Distribution of patients with and without an OI across AHI categories based on manually revised ApneaLink^TM^ score. OI, occupational injury; w/o, without; AHI, apnea-hypopnea index; CI, confidence interval.

Pearson's chi-Square tests were performed to determine whether patients with and without an OI were distributed differently across AHI categories (AHI <5, AHI 5–15, or AHI >15) when using the ApneaLink^TM^ autoscore and after manual revision. Figs 4 and 5 show that the ApneaLink^TM^ autoscore (*p* = 0.337, Fig 4), and manually revised score (*p* = 0.959, Fig 5) did not reveal significant differences in AHI categories between the two groups.

## Discussion

In this cross-sectional study, we investigated the potential of a portable multichannel screening device (ApneaLink^TM^) in diagnosing OSAS among hospital outpatients not referred for sleep apnea investigation. ApneaLink^TM^ results were compared with the gold standard (full-night attended PSG). Furthermore, we evaluated if manual revision of ApneaLink^TM^ data contributed additional benefit in terms of diagnostic performance.

Manual revision of ApneaLink^TM^ data did not appear to contribute an additional diagnostic benefit for patients with a low-to-medium pretest risk for OSAS. The results showed strong correlations for AHI and ODI between ApneaLink^TM^ and PSG. In this study the diagnostic performance of ApneaLink^TM^ appears to be suitable for people with a low-to-medium pretest risk for an OSAS; however, AASM recommends the use of home sleep apnea testing (HSAT) only in patients with a high pre-test probability for OSAS. In addition, a single negative HSAT cannot exclude a diagnosis of OSAS especially if daytime sleepiness, other co-morbidities that are associated with OSAS, or repeated microsleep events are present. Therefore, HSAT does currently not have the potential to replace PSG in this population of patients. When OSAS is suspected either with a low or high AHI in ApneaLink^TM^ testing, patients should undergo investigation with full-night attended PSG.

### Participants' demographic and clinical characteristics

We analyzed a population with a low-to-medium pretest risk for OSAS (mean BMI 26 kg/m^2^, mean age 40 years). Overall, we had a relatively low participation rate (70% of all patients recruited) of patients who spent one night with the ApneaLink^TM^ at home and one night in the sleep laboratory for PSG examination. However, another study reported 79% of valid ApneaLink^TM^ autoscore recordings [37], which is comparable with our study.

In 29% of participating patients investigation in the sleep laboratory was not possible, because the minimum ApneaLink^TM^ recording time (180 min) was not achieved or data were lost due to server problems. The main reason for limited PSG examination was that we were unable to find appropriate dates for some participants as the number of available slots for this study were limited. Moreover, 1% of patients were excluded retrospectively because they were under eighteen years old.

Most studies that investigated the diagnostic performance of ApneaLink^TM^ selected populations with a high pretest risk for OSAS (BMI 30.4 ± 5.3 and 29.3 ± 5.4 kg/m^2^) [38,39]. A recent systematic review and meta-analysis showed that workers suffering from OSAS have a risk of experiencing work accidents nearly double that of control subjects [40]. Therefore, we considered the group of workers with OSAS as having at least a moderate risk for an OI.

AASM recommends that a HSAT should only be used for screening a group of patients with a high pre-test probability for OSAS. In our study, it was the intention to investigate another population in order to find further reasons for more research at best. In addition, it has to be mentioned that a single negative HSAT cannot exclude a diagnosis of OSAS.

### Number of evaluable measurements

A previous study stated that a recording time of less than 4 h when measuring only nasal flow leads to false-negative results (valid for AHI levels of ≥15) [41]. Therefore, we evaluated a minimum of 180 min recorded data from the ApneaLink^TM^ three-channel screening device and analyzed data (which probably contained all stages of sleep as the first REM episode normally occurs about 70 min after falling asleep) [42] to increase comparability with PSG. The mean recording time of 410 min for pulse oximetry and 373 min for nasal flow, allowed us to investigate ApneaLink^TM^ performance in similar circumstances to other studies [38, 41].

When investigating the relationship between AHI and ODI measured by ApneaLink^TM^ compared with PSG, we detected strong to very strong correlations. ApneaLink^TM^ AHI and ODI (autoscore and after manual revision) did not differ significantly from PSG results. In daily clinical practice, often only pulse oximetry is used. Therefore, we also verified if ApneaLink^TM^ ODI correlated strongly with ODI and AHI derived by PSG.

There were two outliers when comparing ApneaLink^TM^ and PSG results: both were men with an OI. In one participant (age 55 years, BMI 28.4 kg/m^2^), AHI derived from the ApneaLink^TM^ was 43 (56 after manual revision) and 5 measured with PSG. The ApneaLink^TM^ recording time was 490 min (all channels). The ApneaLink^TM^ ODI showed 24 desaturations per hour (21 after manual revision), whereas only four desaturations per hour were detected by PSG. The second outlier (age 23 years, BMI 26.8 kg/m^2^) also had a sufficient ApneaLink^TM^ recording time (280 min in all channels). The ApneaLink^TM^ AHI was 1 (2 after manual revision), whereas the AHI obtained with PSG was 56. The ApneaLink^TM^ ODI revealed two desaturations per hour (one after manual revision), whereas 29 desaturations per hour were found with PSG. It is possible that events were scored with ApneaLink^TM^ that would not be scored with PSG (e.g., events not linked to arousal or desaturation). Furthermore, ApneaLink^TM^ is not able to detect whether the events are scored during a period of wakefulness. Other disturbing factors could be displacement of the nasal cannula, damaged ApneaLink^TM^ channels, or nasal obstruction (e.g., due to a cold). Both outliers did not take drugs, which would have reduce the respiratory rate dramatically.

Previous studies with comparable screening devices (not only ApneaLink^TM^) stated that manual revision is useful [37,43], whereas other studies reported that revised results did not differ significantly from autoscores [44]. Our manual evaluator worked without knowing PSG results or the patients' group assignment. A recent study indicated that a nonexpert observer (with 1 year of experience) showed very good agreement with an expert observer on the manual correction of the ApneaLink^TM^ autoscoring [45].

### Correlations between ApneaLink^TM^ and PSG

In Bland Altman plots, we detected that the ApneaLink^TM^ autoscore respectively the ApneaLink^TM^ manually revised score compared to PSG showed random fluctuations, which visibly increased in areas where the mean of AHI was higher than 20. In our two plots, the limits ranged between +/- 14 to 19 units. From a clinical perspective, this is a large and therefore unacceptable spread for the AHI. We therefore have to note that the accuracy of the ApneaLink device is questionable due to the high misclassification rate compared to the PSG and the large spread in the Bland Altman plots.

### Sensitivity, specificity, PPV, and NPV of ApneaLink^TM^

The sensitivity (varied from 12% to 82%) in our study was comparable with other studies (from 67% an an AHI cutoff ≥15 [39] to 91% at an AHI cutoff ≥15 [41]) in which home-measured ApneaLink^TM^ investigation was performed. Again, in these studies, the pretest risk for OSAS was high. The highest sensitivity of ApneaLink^TM^ in our study (82%) was found when comparing AHI≥ 5 with the PSG AHI and/or ODI cutoff ≥15. We consider this result as sufficient because it shows that ApneaLink^TM^ had good sensitivity to detect people with a moderate sleep apnea syndrome. However, interpretation must be with caution when the ApneaLink^TM^ AHI is low in individuals with risk factors for OSAS such as severe snoring, obesity, advanced age, and daytime sleepiness. In cases of uncertainty, PSG is still strongly recommended.

Moreover, the AASM (2015 update) still considers treatment of mild OSAS as optional, because treatment data are based on limited evidence [46]. The highest specificity (99%) for ApneaLink^TM^ in our study was for AHI and/or ODI ≥15 compared with PSG AHI and/or ODI ≥15. Here we can also assume that those who were identified as not having a moderate OSAS were correctly identified.

Manual revision of ApneaLink^TM^ data did not yield relevant clinical benefits. Although there were nine patients for whom manually revised AHI better reflected the AHI obtained from PSG, there were also nine patients who showed a closer association between ApneaLink^TM^ autoscore and PSG results. Moreover, only four patients in the ApneaLink^TM^ investigation (six after manual revision) and 11 patients in the PSG examination had an AHI ≥15. The meaningfulness of the results regarding the AHI cutoff ≥15 should therefore be interpreted with caution. In other studies, the performance of ApneaLink^TM^ also showed very good sensitivity for patients with AHI ≥15 (94%) [38] and 100% for patients with an AHI ≥30 [1].

In addition, we saw that the LR+ was greater than 1 in all analyzed categories (AHI ≥5 and AHI ≥15) of the ApneaLink^TM^ test. The LR+ could help us better interpreting the results of the diagnostic test and shows the approximate change in probability for the disease. The effects on posttest probability of disease varied in our study from modest increase (LR+ >3 for AHI ≥5 ApneaLink^TM^ autoscore and manually revised) to large increase (LR+ >18 for AHI ≥15 ApneaLink^TM^ autoscore respectively LR+ >13 for manually revised score) compared to PSG. Ideally a LR+ should be greater than 20 or at least 10 to be considered excellent or good. A LR+ of 3 for OSAS is indicative of a poor test.

### Comparison of AHI between ApneaLink^TM^ (autoscore and manually revised) and PSG

We demonstrated (Figs 4 and 5) that it was not possible to show that patients with an OI suffer more from OSAS than patients without an OI using ApneaLink^TM^. PSG investigation is therefore still needed. As reported in other studies, workers with a diagnosed OSAS have a higher risk for experiencing workplace accident [40, 47].

### Strengths and limitations of this study/ApneaLink^TM^

This study was conducted in a real-life setting using a population that is not normally screened for OSAS. Patients in our sample had no medical assignment and no Epworth Sleepiness Score reflecting daytime sleepiness. Moreover, we used PSG (the gold standard for OSAS diagnosis), which is expensive and complex, to control ApneaLink^TM^ performance. ApneaLink^TM^ avoids observer bias by interpreting the recordings through the ApneaLink^TM^ software itself.

In contrast to the positive features of ApneaLink^TM^, certain weaknesses need to be mentioned. ApneaLink^TM^ is not able to distinguish between central and obstructive sleep apneas [36]. If the breathing tube is kinked or has clogs, the sensor unit is defective, or the tube is not connected correctly, the signal is either weakened or invalidated. The finger pulse sensor can also be released from the finger during the night, affecting the recording process. Moreover, pulse sensor results can be falsified by nail varnish or acrylic fingernails [48], restricted peripheral capillary circulation, or nails affected by yeasts or filamentous fungi [49].

ApneaLink^TM^ data were recorded at home by ambulant patients, after instruction by a technical assistant at the hospital. However, there was no control regarding whether ApneaLink^TM^ was worn correctly. Recording a second measurement at home might have provided more accurate data as a second measurement has been shown to have additional benefit when the AHI cutoff is ≥15 [39].

It must be noted that newer ApneaLink^TM^ devices, which also measure the effort, are nowadays available for HSAT. However, during data collection these devices were not yet available and therefore the correlation of newer ApneaLink^TM^ devices with PSG may be different. No sleep diaries were obtained during ApneaLink^TM^ testing to record the subjective quality and duration of sleep. It must be mentioned that measurement with the ApneaLInk^TM^ was performed in the night after the ER visit and sleep disruption due to pain from the injury cannot be excluded. None of the study participants was under treatment with high-potent opioid analgesics during the sleep studies, but it is possible that exposure to acetaminophene, NSAID or low-potent opioids like codein was different during the two sleep studies.

There were 2 individuals that did not fulfill the inclusion criteria as they were younger than 18 years, and their datasets were not included in the analysis.

Questionable is also the performance of the manual revision if it is reaplicable in the same manner. Despite revising all apnea, hypopnea and desaturations of 15 nights recorded by the ApneaLink^TM^ step by step together with the head of the sleep laboratory according to preinstalled definitions of the ApneaLink^TM^ device (also accepted from AASM), the quality of the revision may probably not be the same as if performed by an expert, although feedback was always demanded in case of unclear events. However, the study of Nigro et al. [45], which mentioned that a non-expert observer (with 1 year of experience) had very good agreement with an expert observer on the manual correction of the ApneaLink^TM^ autoscore, encouraged us to do so.

## Conclusions

Our results show that ApneaLink^TM^ may be used as a clinical screening device for OSAS in patients with an OI. However, given the low number of study participants with OSAS in our study and the AASM recommendation for the use of HSAT only in patients with a high pre-test probability, we do not suggest that HSAT can replace full-night attended PSG in every case. Nevertheless, when compared with data obtained in the sleep laboratory, ApneaLink^TM^ (autoscore and after manual revision) demonstrated good sensitivity and specificity (82%) when using a AHI threshold ≥5 to detect moderate-to-severe OSAS (AHI and/or ODI ≥15) as derived by full-night attended PSG.This emphasizes the capacity of ApneaLink^TM^ to recognize OSAS. However, the accuracy of the ApneaLink device remains questionable due to the high misclassification rate compared to the PSG and the large spread in the Bland Altman plots.

Considering our results, automatic scoring from ApneaLink^TM^ may be used to have an idea if an OSAS in patients with a low pretest risk might be present or not. However, to send people with a low-to-medium pretest risk for OSAS to PSG without clinical symptoms is inconceivable. Although ApneaLink^TM^ is a significantly cheaper alternative to PSG for OSAS diagnostics, PSG investigation is still indicated in uncertain cases to clearly exclude an OSAS, depending on the AHI cutoff defined by the clinician. This can be the case if patients have risk factors for OSAS and present with excessive daytime sleepiness. In cases of uncertainty, ApneaLink^TM^ investigation could be repeated before conducting a full-night attended PSG to substatiate the suspicion having an OSAS. We also recommend that issues such as marital problems, loss of job, or experiencing a traffic accident are not disregarded, as these problems may occur simultaneously and might be complications of an OSAS [50]. The use of the automatic ApneaLink^TM^ device requires development of integrated networks between nonspecialist and specialist practitioners to allow achieving an expert system to be managed by nonexperts [37].

## Supporting information

S1 Dataset (XLSX)

## References

[1] Koyama RG, Esteves AM, Oliveira e Silva L, Lira FS, Bittencourt LR, Tufik S, et al Prevalence of and risk factors for obstructive sleep apnea syndrome in Brazilian railroad workers. Sleep Med. 2012;13(8):1028–32. doi: 10.1016/j.sleep.2012.06.017 22841037 10.1016/j.sleep.2012.06.017

[2] Stuck BA, Maurer JT, Schredl M, Weess HG. Praxis der Schlafmedizin. 2nd ed. Berlin, Heidelberg: Springer Berlin Heidelberg; 2013.

[3] Tishler PV, Larkin EK, Schluchter MD, Redline S. Incidence of sleep-disordered breathing in an urban adult population: the relative importance of risk factors in the development of sleep-disordered breathing. Jama. 2003;289(17):2230–7. doi: 10.1001/jama.289.17.2230 12734134 10.1001/jama.289.17.2230

[4] de Silva S, Abeyratne U, Hukins C. Gender dependant snore sound based multi feature obstructive sleep apnea screening method. Conference proceedings: Annual International Conference of the IEEE Engineering in Medicine and Biology Society IEEE Engineering in Medicine and Biology Society Annual Conference. 2012;2012:6353–6. 10.1109/EMBC.2012.6347447 23367382

[5] Young T, Palta M, Dempsey J, Skatrud J, Weber S, Badr S. The occurrence of sleep-disordered breathing among middle-aged adults. The New England journal of medicine. 1993;328(17):1230–5. doi: 10.1056/NEJM199304293281704 8464434 10.1056/NEJM199304293281704

[6] Bearpark H, Elliott L, Grunstein R, Cullen S, Schneider H, Althaus W, et al Snoring and sleep apnea. A population study in Australian men. American journal of respiratory and critical care medicine. 1995;151(5):1459–65. doi: 10.1164/ajrccm.151.5.7735600 7735600 10.1164/ajrccm.151.5.7735600

[7] Sleep-related breathing disorders in adults: recommendations for syndrome definition and measurement techniques in clinical research. The Report of an American Academy of Sleep Medicine Task Force. Sleep. 1999;22(5):667–89. 10450601

[8] Bixler EO, Kales A, Soldatos CR, Kales JD, Healey S. Prevalence of sleep disorders in the Los Angeles metropolitan area. The American journal of psychiatry. 1979;136(10):1257–62. doi: 10.1176/ajp.136.10.1257 314756 10.1176/ajp.136.10.1257

[9] Guilleminault C, van den Hoed J, Mitler MM. Clinical overview of the sleep apnea syndromes Guilleminault C, Dement WC, editors. New York: Alan R. Liss; 1978 1–12 p.

[10] Janson C, Hillerdal G, Larsson L, Hultcrantz E, Lindholm CE, Bengtsson H, et al Excessive daytime sleepiness and fatigue in nonapnoeic snorers: improvement after UPPP. The European respiratory journal. 1994;7(5):845–9. 8050539

[11] Ulfberg J, Carter N, Talback M, Edling C. Excessive daytime sleepiness at work and subjective work performance in the general population and among heavy snorers and patients with obstructive sleep apnea. Chest. 1996;110(3):659–63. 8797408 10.1378/chest.110.3.659

[12] Javaheri S, Parker TJ, Liming JD, Corbett WS, Nishiyama H, Wexler L, et al Sleep apnea in 81 ambulatory male patients with stable heart failure. Types and their prevalences, consequences, and presentations. Circulation. 1998;97(21):2154–9. 9626176 10.1161/01.cir.97.21.2154

[13] Sin DD, Fitzgerald F, Parker JD, Newton G, Floras JS, Bradley TD. Risk factors for central and obstructive sleep apnea in 450 men and women with congestive heart failure. American journal of respiratory and critical care medicine. 1999;160(4):1101–6. doi: 10.1164/ajrccm.160.4.9903020 10508793 10.1164/ajrccm.160.4.9903020

[14] Robinson GV, Stradling JR, Davies RJ. Sleep. 6: obstructive sleep apnoea/hypopnoea syndrome and hypertension. Thorax. 2004;59(12):1089–94. doi: 10.1136/thx.2003.015875 15563710 10.1136/thx.2003.015875 PMC1746904

[15] Peppard PE, Young T, Palta M, Skatrud J. Prospective study of the association between sleep-disordered breathing and hypertension. The New England journal of medicine. 2000;342(19):1378–84. doi: 10.1056/NEJM200005113421901 10805822 10.1056/NEJM200005113421901

[16] Shahar E, Whitney CW, Redline S, Lee ET, Newman AB, Nieto FJ, et al Sleep-disordered breathing and cardiovascular disease: cross-sectional results of the Sleep Heart Health Study. American journal of respiratory and critical care medicine. 2001;163(1):19–25. doi: 10.1164/ajrccm.163.1.2001008 11208620 10.1164/ajrccm.163.1.2001008

[17] Hamilton GS, Solin P, Naughton MT. Obstructive sleep apnoea and cardiovascular disease. Internal medicine journal. 2004;34(7):420–6. doi: 10.1111/j.1445-5994.2004.00596.x 15271177 10.1111/j.1445-5994.2004.00596.x

[18] Bassetti C, Aldrich MS. Sleep apnea in acute cerebrovascular diseases: final report on 128 patients. Sleep. 1999;22(2):217–23. 10201066 10.1093/sleep/22.2.217

[19] Ip MS, Lam B, Ng MM, Lam WK, Tsang KW, Lam KS. Obstructive sleep apnea is independently associated with insulin resistance. American journal of respiratory and critical care medicine. 2002;165(5):670–6. doi: 10.1164/ajrccm.165.5.2103001 11874812 10.1164/ajrccm.165.5.2103001

[20] Punjabi NM, Sorkin JD, Katzel LI, Goldberg AP, Schwartz AR, Smith PL. Sleep-disordered breathing and insulin resistance in middle-aged and overweight men. American journal of respiratory and critical care medicine. 2002;165(5):677–82. doi: 10.1164/ajrccm.165.5.2104087 11874813 10.1164/ajrccm.165.5.2104087

[21] Kapur VK, Auckley DH, Chowdhuri S, Kuhlmann DC, Mehra R, Ramar K, et al Clinical Practice Guideline for Diagnostic Testing for Adult Obstructive Sleep Apnea: An American Academy of Sleep Medicine Clinical Practice Guideline. Journal of clinical sleep medicine: JCSM: official publication of the American Academy of Sleep Medicine. 2017;13(3):479–504. 28162150 10.5664/jcsm.6506 PMC5337595

[22] Flemons WW, Douglas NJ, Kuna ST, Rodenstein DO, Wheatley J. Access to diagnosis and treatment of patients with suspected sleep apnea. American journal of respiratory and critical care medicine. 2004;169(6):668–72. doi: 10.1164/rccm.200308-1124PP 15003950 10.1164/rccm.200308-1124PP

[23] Young T, Evans L, Finn L, Palta M. Estimation of the clinically diagnosed proportion of sleep apnea syndrome in middle-aged men and women. Sleep. 1997;20(9):705–6. 9406321 10.1093/sleep/20.9.705

[24] Teran Santos J, Moreno G, Rodenstein DO. [Sleep medicine and transport workers. Medico-social aspects with special reference to sleep apnoea syndrome]. Archivos de bronconeumologia. 2010;46(3):143–7. doi: 10.1016/j.arbres.2009.08.004 19815327 10.1016/j.arbres.2009.08.004

[25] Horne JA, Reyner LA. Sleep related vehicle accidents. BMJ (Clinical research ed). 1995;310(6979):565–7. 10.1136/bmj.310.6979.565 PMC2548939 7888930

[26] Gauchard GC, Chau N, Touron C, Benamghar L, Dehaene D, Perrin P, et al Individual characteristics in occupational accidents due to imbalance: a case-control study of the employees of a railway company. Occupational and environmental medicine. 2003;60(5):330–5. doi: 10.1136/oem.60.5.330 12709517 10.1136/oem.60.5.330 PMC1740530

[27] Overstall PW, Johnson AL, Exton-Smith AN. Instability and falls in the elderly. Age and ageing. 1978;Suppl:92–6. 727067 10.1093/ageing/7.suppl.92

[28] Perrin PP, Jeandel C, Perrin CA, Bene MC. Influence of visual control, conduction, and central integration on static and dynamic balance in healthy older adults. Gerontology. 1997;43(4):223–31. doi: 10.1159/000213854 9222751 10.1159/000213854

[29] Nardone A, Tarantola J, Giordano A, Schieppati M. Fatigue effects on body balance. Electroencephalography and clinical neurophysiology. 1997;105(4):309–20. 9284239 10.1016/s0924-980x(97)00040-4

[30] Johnston RB 3rd, Howard ME, Cawley PW, Losse GM. Effect of lower extremity muscular fatigue on motor control performance. Medicine and science in sports and exercise. 1998;30(12):1703–7. 9861603 10.1097/00005768-199812000-00008

[31] Nelson HD, Nevitt MC, Scott JC, Stone KL, Cummings SR. Smoking, alcohol, and neuromuscular and physical function of older women. Study of Osteoporotic Fractures Research Group. Jama. 1994;272(23):1825–31. 7990216 10.1001/jama.1994.03520230035035

[32] Iki M, Ishizaki H, Aalto H, Starck J, Pyykko I. Smoking habits and postural stability. American journal of otolaryngology. 1994;15(2):124–8. 8179103 10.1016/0196-0709(94)90061-2

[33] Pereira CB, Strupp M, Holzleitner T, Brandt T. Smoking and balance: correlation of nicotine-induced nystagmus and postural body sway. Neuroreport. 2001;12(6):1223–6. 11338195 10.1097/00001756-200105080-00033

[34] Schlesinger A, Redfern MS, Dahl RE, Jennings JR. Postural control, attention and sleep deprivation. Neuroreport. 1998;9(1):49–52. 9592046 10.1097/00001756-199801050-00010

[35] Kitamura T, Yoshida M, Morimoto Y, Narui K, Tsuda T, Kikuchi H, et al [Surveillance of industrial physicians' knowledge and concern about sleep apnea syndrome]. Nihon Jibiinkoka Gakkai kaiho. 2005;108(1):20–6. 15712493 10.3950/jibiinkoka.108.20

[36] Schweiz R. ResMed ApneaLinkTM Broschüre 2006. Available from: http://www.vivisol.at/assets/uploads/services_products/ApneaLink_Brosch%C3%BCre_d.pdf.

[37] Masa JF, Duran-Cantolla J, Capote F, Cabello M, Abad J, Garcia-Rio F, et al Effectiveness of home single-channel nasal pressure for sleep apnea diagnosis. Sleep. 2014;37(12):1953–61. doi: 10.5665/sleep.4248 25325484 10.5665/sleep.4248 PMC4237536

[38] Nigro CA, Serrano F, Aimaretti S, Gonzalez S, Codinardo C, Rhodius E. Utility of ApneaLink for the diagnosis of sleep apnea-hypopnea syndrome. Medicina. 2010;70(1):53–9. 20228025

[39] Crowley KE, Rajaratnam SM, Shea SA, Epstein LJ, Czeisler CA, Lockley SW. Evaluation of a single-channel nasal pressure device to assess obstructive sleep apnea risk in laboratory and home environments. Journal of clinical sleep medicine: JCSM: official publication of the American Academy of Sleep Medicine. 2013;9(2):109–16. 23372462 10.5664/jcsm.2400 PMC3544377

[40] Garbarino S, Guglielmi O, Sanna A, Mancardi GL, Magnavita N. Risk of Occupational Accidents in Workers with Obstructive Sleep Apnea: Systematic Review and Meta-analysis. Sleep. 2016;39(6):1211–8. doi: 10.5665/sleep.5834 26951401 10.5665/sleep.5834 PMC4863208

[41] Erman MK, Stewart D, Einhorn D, Gordon N, Casal E. Validation of the ApneaLink for the screening of sleep apnea: a novel and simple single-channel recording device. Journal of clinical sleep medicine: JCSM: official publication of the American Academy of Sleep Medicine. 2007;3(4):387–92. 17694728 PMC1978315

[42] McCarley RW. Neurobiology of REM and NREM sleep. Sleep Med. 2007;8(4):302–30. doi: 10.1016/j.sleep.2007.03.005 17468046 10.1016/j.sleep.2007.03.005

[43] Nigro CA, Dibur E, Malnis S, Grandval S, Nogueira F. Validation of ApneaLink Ox for the diagnosis of obstructive sleep apnea. Sleep & breathing = Schlaf & Atmung. 2013;17(1):259–66. 22447171 10.1007/s11325-012-0684-4

[44] Lesser DJ, Haddad GG, Bush RA, Pian MS. The utility of a portable recording device for screening of obstructive sleep apnea in obese adolescents. Journal of clinical sleep medicine: JCSM: official publication of the American Academy of Sleep Medicine. 2012;8(3):271–7. 22701384 10.5664/jcsm.1912 PMC3365085

[45] Nigro CA, Malnis S, Dibur E, Rhodius E. How reliable is the manual correction of the autoscoring of a level IV sleep study (ApneaLink) by an observer without experience in polysomnography? Sleep & breathing = Schlaf & Atmung. 2012;16(2):275–9. 21537906 10.1007/s11325-011-0524-y

[46] Ramar K, Dort LC, Katz SG, Lettieri CJ, Harrod CG, Thomas SM, et al Clinical Practice Guideline for the Treatment of Obstructive Sleep Apnea and Snoring with Oral Appliance Therapy: An Update for 2015. Journal of clinical sleep medicine: JCSM: official publication of the American Academy of Sleep Medicine. 2015;11(7):773–827. 26094920 10.5664/jcsm.4858 PMC4481062

[47] Hirsch Allen AJ, Park JE, Daniele PR, Fleetham J, Ryan CF, Ayas NT. Obstructive sleep apnoea and frequency of occupational injury. Thorax. 2016;71(7):664–6. doi: 10.1136/thoraxjnl-2015-207994 26980010 10.1136/thoraxjnl-2015-207994

[48] Hinkelbein J, Koehler H, Genzwuerker HV, Fiedler F. Artificial acrylic finger nails may alter pulse oximetry measurement. Resuscitation. 2007;74(1):75–82. doi: 10.1016/j.resuscitation.2006.11.018 17353079 10.1016/j.resuscitation.2006.11.018

[49] Heck M, Fresenius M. Repetitorium Anästhesiologie. 5th ed. Berlin, Heidelberg: Springer Berlin Heidelberg; 2007.

[50] Laube I, Seeger R, Russi EW, Bloch KE. Accidents related to sleepiness: review of medical causes and prevention with special reference to Switzerland. Schweizerische medizinische Wochenschrift. 1998;128(40):1487–99. 9888163

